# Supplementary material for: Giant Huang–Rhys Factor for Electron Capture by the Iodine Intersitial in Perovskite Solar Cells
Source: J Am Chem Soc. 2021 Jun 9;143(24):9123–8. doi: 10.1021/jacs.1c03064 (PMC8297730; doi:10.1021/jacs.1c03064)
Supplement: Supplementary file 1 — ja1c03064_si_001.pdf [file ja1c03064_si_001.pdf]

Supporting Information: Giant Huang-Rhys Factor for Electron Capture by the Iodine Intersitial in Perovskite Solar Cells

Lucy D. Whalley,[*,†] Puck van Gerwen,[‡] Jarvist M. Frost,[¶] Sunghyun Kim,[‡] Samantha N. Hood,[‡] and Aron Walsh[*,‡,§]

†*Department of Mathematics, Physics and Electrical Engineering, Northumbria University, Newcastle upon Tyne, NE1 8QH, UK*

‡*Department of Materials, Imperial College London, London SW7 2AZ, UK*

¶*Department of Physics, Imperial College London, London SW7 2AZ, UK*

§*Department of Materials Science and Engineering, Yonsei University, Seoul 03722, Korea*

E-mail: l.whalley@northumbria.ac.uk; a.walsh@imperial.ac.uk

May 26, 2021

Data access

The following data and analysis scripts are available online at https://github.com/lucydot/MAPI_iodine_vibrations:

- crystal structures for the iodine interstitial in the negative, neutral and positive charge states
- harmonic phonon data at the gamma point, including the eigenfunctions, frequencies and inverse participation ratios

- a Jupyter Notebook with additional analysis of the harmonic phonon data

Further details of the calculation procedure for defect properties

Geometry relaxation procedure: So that no preference was given to a particular combination of octahedral tilts the starting point for atomic relaxation was MAPI in the pseudo-cubic phase. Upon adding an iodine interstitial the structure was found to have a number of local minima and multiple relaxations between charge states were required to reach a global minimum. To calculate the collective atomic displacement (ΔQ) between charge states accurately the starting point for relaxation of the charged states must be the neutral charge state geometry. The atomic relaxation procedure is outlined in Figure S1.

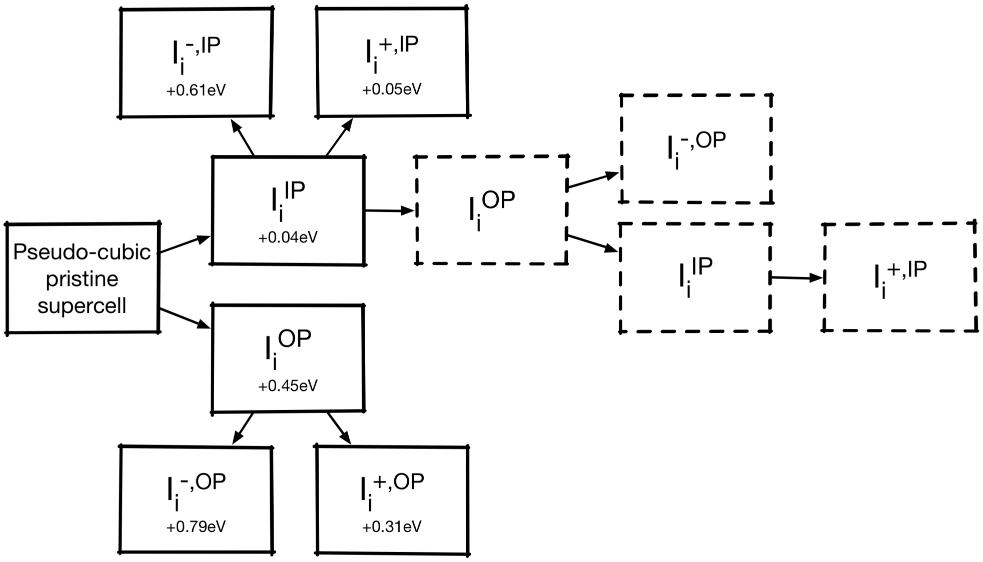

Figure S1: Atomic relaxation procedure for point defects in hybrid halide perovskites in the pseudo-cubic phase. IP indicates that the defect is lying in the *ab*-plane, and OP indicates that the defect is lying along the *c*-axis. The lowest energy structures are in a dash-line box. The hybrid halide perovskite structure has a number of local minima and multiple relaxations were required to break symmetry and reach a global minimum. For the higher energy defect structures the energy above the global minimum for that charge state is given.

The neutral iodine interstitial I_i relaxes to two defect geometries (see Figure 3 in the main

text). These structures differ by only 0.04 eV in energy. The lowest energy structure contains a I_2^- split interstitial that lies out-of-plane (along the c-axis) with bond length 3.19 Å. This defect is denoted I_i^{OP}. The higher energy structure contains a I_2^- split interstitial that lies in the ab-plane with bond length 3.24 Å. This is denoted I_i^{IP}. The negative iodine interstitial I_i^- relaxes to an out-of-plane position along the c-axis. I_i^- forms a split interstitial around a lattice iodine site with two independently coordinated iodine ions symmetrically bridging two lead atoms. The I–I distance is 3.82 Å. As I_i^- lies out-of-plane, we expect potential charge trapping processes to occur between I_i^- and I_i^{OP}. The positive interstitial forms an in-plane trimer structure with bond lengths 2.90 Å and 2.96 Å. As I_i^+ lies in-plane, we expect potential charge trapping processes to occur between I_i^+ and I_i^{IP}. Table S1 contains a summary of the defect geometries and a comparison to values in the literature.

Table S1: Defect orientation and bond length of I_i^+,I_i^-,I_i^{IP} and I_i^{OP} in MAPI, with a comparison to computational results in the literature. The bond lengths are given in Å.

	This work		Meggiolaro et al., Ref.[S1]		Du, Ref.[S2]
	orientation	I–I bond length	orientation	I–I bond length	orientation
I_i^-	out-of-plane	3.82	out-of-plane	3.89	in-plane
I_i^+	in-plane	2.89/2.95	in-plane	2.95 average	in-plane
I_i^{OP}	out-of-plane	3.19	out-of-plane	3.24	-
I_i^{IP}	in-plane	3.24	in-plane	3.88	-

Defect formation energy: The formation energy of a defect in charge state q is given by

$$E_{\mathrm{f}}(q) = E_{\mathrm{d}}(q) - E_{\mathrm{b}} - \sum_i \mu_i n_i + q(\epsilon_{\mathrm{VBM}} + E_{\mathrm{F}}) + E_{\mathrm{corr}}, \tag{S1}$$

where $E_{\mathrm{d}}(q)$ is the total energy of the supercell, E_{b} is the total energy of the pristine lattice, μ_i is the chemical potential of species i and n_i is the number of atoms that are added or removed. $E_{\mathrm{d}}(q)$, E_{b} and μ_i were calculated using DFT, as outlined in the previous section. The defect correction E_{corr} consists of two terms – the image charge correction (calculated using Ref.[S3]) for charged defects and a tilting correction that is specific to pseudo-cubic perovskites.

The static dielectric constant of MAPI is large ($\bar{\epsilon}_0 = 22.67$)[S4] and so the lattice can

effectively screen charged defects, in addition the point defects in this study have a maximum charge of one and the supercell is relatively large (193 atoms). These factors lead to a small image charge correction of $-0.057\,\mathrm{eV}$.

All of the defect formation energies are referenced to the minimum energy of the pristine lattice. However the geometry of the pristine lattice in the pseudo-cubic phase corresponds to a time average and is not the minimum energy structure (Figure S2). To calculate the minimum energy of the lattice the pseudo-cubic structure can be distorted along the soft mode at the R-point in q-space. This 'modemapping' procedure[S5] is not compatible with the supercell expansion used for the 197-atom supercell, but the correction energy can be inferred from modemapping other supercell expansions (Table S2).

Note that in our model we do not consider possible finite-temperature fluctuations in the trap energy. In the case of the bromine vacancy in hybrid perovskite this leads to shifts in trap energy up to $1\,\mathrm{eV}$.[S6]

Table S2: Tilting corrections for supercell expansions of the pseudo-cubic perovskite lattice. The 768-atom supercell is eight times larger than the 96-atom supercell, so we expect the tilting correction to be eight times larger. It is calculated to be 7.5 times larger. The tilting correction for the 192-atom expansion, which cannot be calculated directly, is twice as large as the correction for the 96-atom supercell.

supercell expansion	# atoms	tilting correction (meV)
$2 \times 2 \times 2$	96	37 (calculated)
$2\sqrt{2} \times 2\sqrt{2} \times 2$	192	74 (predicted)
$4 \times 4 \times 4$	768	270 (calculated)

Charge transition levels: The calculated charge transition levels are given in Figure S3. As is found in the previous literature[S1,S2] there is negative-U behaviour, where the (+/0) transition level is higher in energy than the (0/-) transition. There is one active charge trapping level in the bandgap, corresponding to electron trapping at I_i^+. The charge transition levels generated using the intermediate higher energy structures (Figures S1 and S4) reproduce the results in the literature, highlighting how sensitive the defect energetcs are the defect geometry.

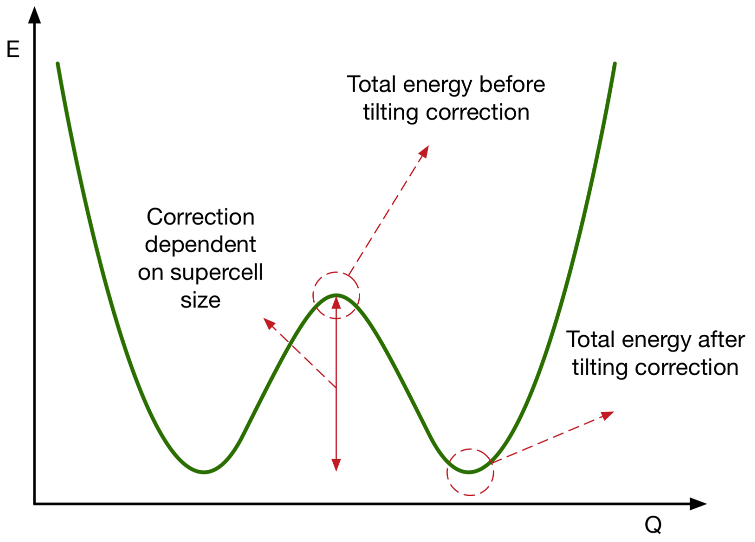

Figure S2: A schematic of the tilting correction. The green line is a double well potential energy surface that is typical of pseudo-cubic perovskite structures. The tilting correction is only needed when using a high-symmetry pseudo-cubic perovskite phase to calculate the pristine bulk energy.

Carrier capture rate: The procedure follows static coupling theory as implemented by Alkauskas et al.[S7] and recently extended to anharmonic potential energy surfaces by Kim et al. in the CARRIERCAPTURE.JL package.[S8,S9] The electron capture coefficient C_n determines the electron capture rate R_n at a neutral defect via the equation

$$R_n = C_n N_0 n, \tag{S2}$$

where N_0 is the neutral defect density and n is the electron density. The capture coefficient is derived by considering electron phonon coupling at first order and using a one-dimensional approximation so that the problem reduces to a single phonon mode Q. As outlined in the main text, for electron capture from an initial state i to a final state f, the carrier capture

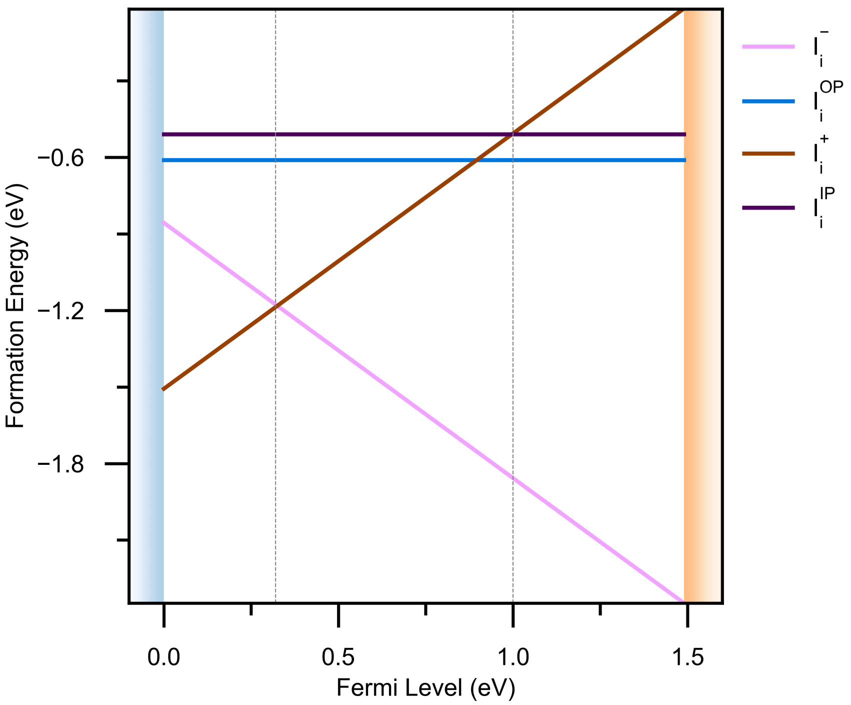

Figure S3: Charge transition levels of the iodine interstitial defect in MAPI, calculated using the hybrid HSE06 funtional with spin-orbit coupling. This assumes iodine rich conditions, a crystal in equilibrium with I_2(g), where interstitial formation is energetically favoured. IP indicates that the defect is lying in the *ab*-plane and OP indicates that the defect is lying along the *c*-axis.

coefficient is given by

$$C_n = V\frac{2\pi}{\hbar}gW_{\text{if}}^2\sum_m \Theta_m \sum_n |\langle\chi_{im}|Q-Q_0|\chi_{fn}\rangle^2 \times \delta(\Delta E + m\hbar\omega_{\text{i}} - n\hbar\omega_{\text{f}}), \tag{S3}$$

where V is the supercell volume, g is the energetic degeneracy of the final state, W_{if} is the electron-phonon coupling matrix element, $\langle\chi_{im}|Q-Q_0|\chi_{fn}\rangle$ is the overlap of the vibrational wavefunctions χ and the Dirac $\delta(\Delta E + m\hbar\omega_{\text{i}} - n\hbar\omega_{\text{f}})$ ensures that there is conservation of energy. In practice the Dirac δ term is replaced by a smearing function; for the calculations in this study this is a gaussian function of width 0.01 eV. Θ_m in Equation S3 is the thermal

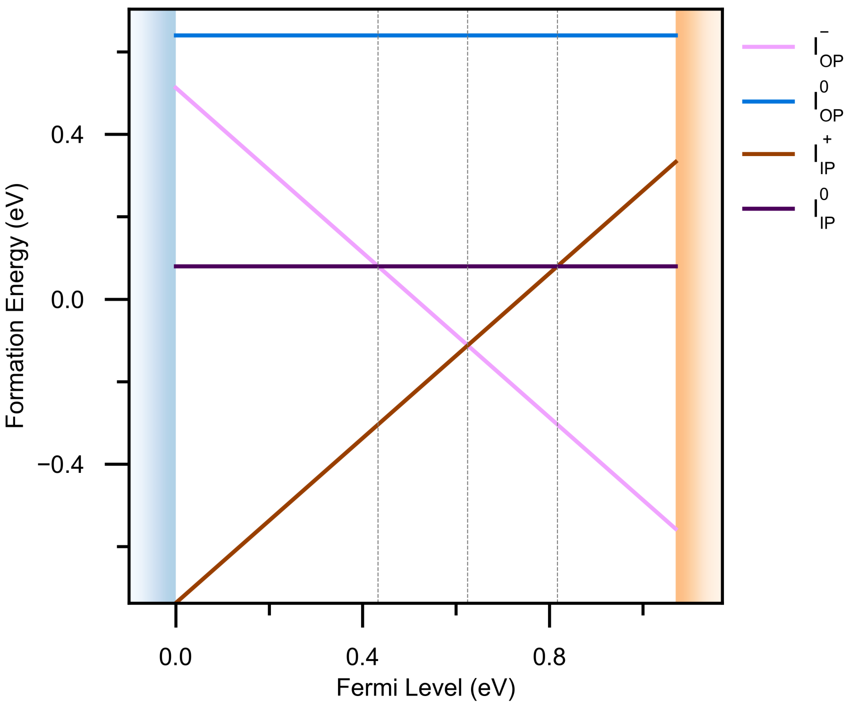

Figure S4: Charge transition levels of the higher energy iodine interstitial defects in MAPI. The defect geometries correspond to those in the solid boxes in Figure S1. Defects with a lower energy were later found and the charge transition diagram for these are reported above.

occupation of the vibrational state m:

$$\Theta_m = \frac{e^{-\frac{m\hbar\omega_m}{k_B T}}}{\sum_m e^{-\frac{m\hbar\omega_m}{k_B T}}}. \tag{S4}$$

The anharmonic potential energy surface was generated from fitting a spline of order four, as implemented in the DIERCKX.JL package,[S10] to the DFT total energies. We consider electron capture from the neutral charge state to the negative charge state which has a singly energy-degenerate geometry.

The 1D Schrödinger equation for the potential energy surface was solved using a finite difference method implemented in the BROOGLIE package[S11] to give the vibrational wave-

functions. Using the one-dimensional approximation discussed above, the electron-phonon coupling can be described with a single matrix element:[S7]

$$W_{\text{if}} = \langle \Psi_{\text{i}} | \frac{\partial \hat{H}}{\partial Q} | \Psi_{\text{f}} \rangle, \tag{S5}$$

where $\Psi_{\{i,f\}}$ are many-electron wavefunctions and $\hat{H}$ is the many-body Hamiltonian of the system. We assume that the many-body Hamiltonian and many-electron wavefunctions can be replaced by their single particle counterparts, so that the electron-phonon coupling matrix element is given by:[S7]

$$W_{\text{if}} = (\epsilon_{\text{f}} - \epsilon_{\text{i}}) \langle \psi_{\text{i}} | \frac{\partial \psi_{\text{f}}}{\partial Q} \rangle, \tag{S6}$$

where the single particle wavefunction of the initial (final) charge state is given by ψ_{i} (ψ_{f}) and has an eigenstate energy of ϵ_{i} (ϵ_{f}). The PAWPYSEED package[S12] was used to derive the orthogonal wavefunctions from the pseudo wavefunctions and perform the overlap integrals in real space. Further details of the methodology can be found in the literature.[S7]

There are several approximations built into the methodology outlined above:

- Static coupling approximation. This assumes that the timescales of the carrier capture process are longer than the phonon lifetimes and periods of lattice vibrations. For this system the iodine interstitial in the neutral charge state has an effective frequency of 1.4 THz, corresponding to a lattice vibration period of the order 10^{-12} s. Using our calculated capture coefficient of $1 \times 10^{-10}\,\text{cm}^3\,\text{s}^{-1}$, and assuming an electron density of $1 \times 10^{15}\,\text{cm}^{-3}$ (typical for a solar cell under one sun illumination) gives a carrier capture timescale of 1×10^{-5} s (at a single point defect).

- The description of electron-phonon coupling to first order in perturbation theory. A perturbative treatment of the charge transition is valid for electron-phonon coupling below a threshold value. With reference to previous results in the literature[S7] the value calculated in this work, $0.0036\,\text{eVamu}^{-1/2}\text{Å}^{-1}$, is within that range.

- Linear coupling approximation. In this approximation the electron-phonon coupling

term is Taylor expanded in Q and only the first order terms are retained.

- Single mode approximation. This relates to the use of a single configuration coordinate Q. Please see the following section for further discussion in the context of this work.

Additional analysis of the carrier capture rate

Electron-phonon coupling term: The inner product of the wavefunctions at the conduction band minimum ϕ_{i} and unoccupied defect level ϕ_{f} are calculated at various Q-values and substituted into Equation S3 to give a value of 0.0036 eVamu$^{-1/2}$Å^{-1} for the electron-phonon coupling matrix element (as reported in the main text).

It is possible to estimate the electron-phonon coupling term using an estimate for the degree of localisation of ϕ_{i} and ϕ_{f}. For strong electron phonon coupling W_{if} corresponds to[S7]

$$W_{\mathrm{if}} \approx \frac{\Delta E}{\Delta Q}\sqrt{\frac{M_d}{M_b}}, \tag{S7}$$

where M_b is the number of atoms in the supercell and M_d is the number of atoms the defect state is localised around. For our system $W_{\mathrm{if}} \approx 0.0048$ and so electron-phonon coupling for the single mode Q is strong.

Classical barrier vs tunnelling: As shown in Figure S5, there is significan quantum tunnelling before the barrier, in both the harmonic and anharmonic case.

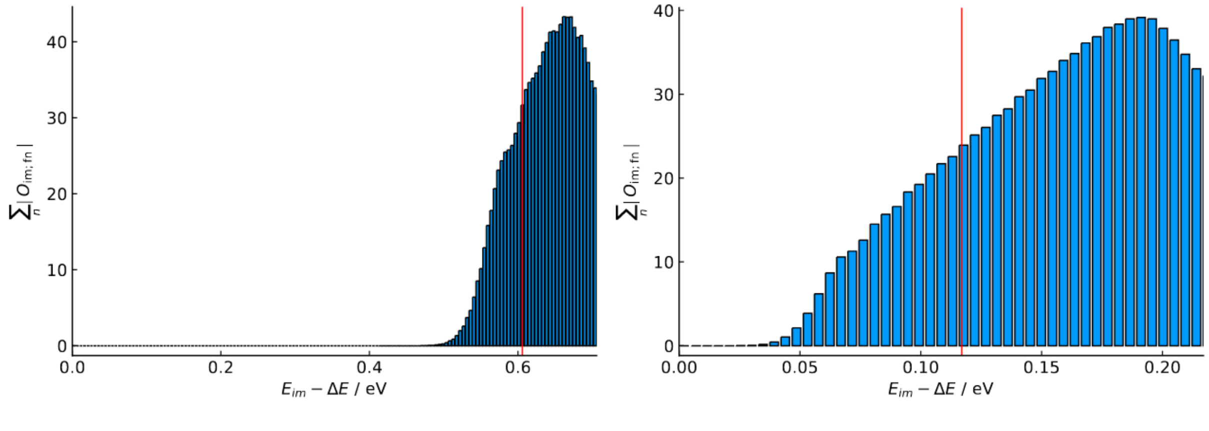

Figure S5: Whether a (a) harmonic or (b) anharmonic description of the PES is used, electron capture by $MAPbI_3$:I_i is not fully classical as phonon overlap $\sum_m O_{im;tn}$ persists before the classical barrier E_b, indicated by a red vertical line.

Additional analysis of defect lattice geometries

As discussed in the main text, analysis of the defect lattice geometries in S6 show that the large lattice relaxation after charge capture is associated with rotations of the inorganic octahedral cage.

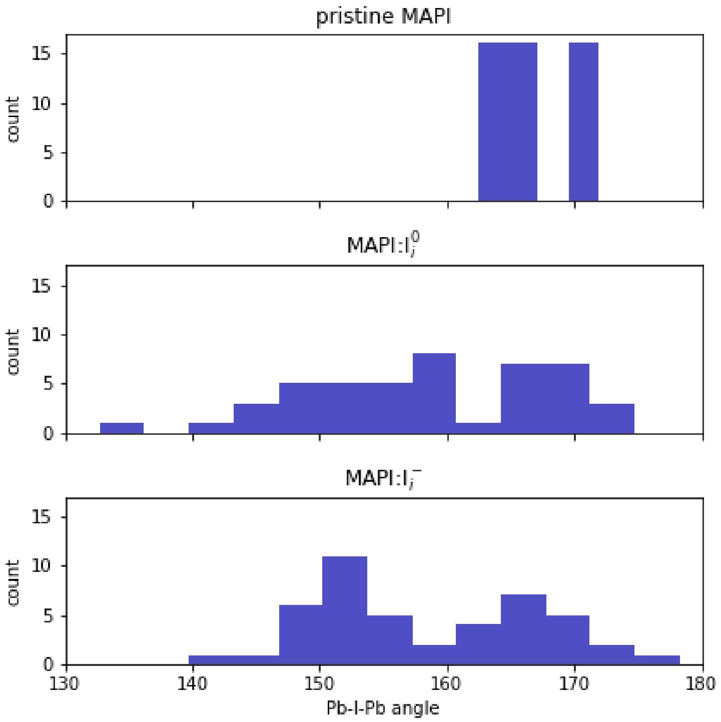

Figure S6: For pristine MAPI in the pseudo-cubic phase(top figure) there are three distinct Pb-I-Pb bond angles. Upon introduction of the neutral iodine interstitial (middle figure) the lattice becomes more disordered and includes a wider distribution of bond angles. After charge capture a more symmetric structure is found (Figure 3 in the main text) and the bond angles shift to form a bimodal distribution.

References

(S1) Meggiolaro, D.; Motti, S. G.; Mosconi, E.; Barker, A. J.; Ball, J.; Andrea Riccardo Perini, C.; Deschler, F.; Petrozza, A.; De Angelis, F. Iodine chemistry determines the defect tolerance of lead-halide perovskites. *Energy Environ.* **2018**, *11*, 702–713.

(S2) Du, M.-H. Density Functional Calculations of Native Defects in $CH_3NH_3PbI_3$: Effects

of Spin–Orbit Coupling and Self-Interaction Error. *J. Phys. Chem. Lett.* **2015**, *6*, 1461–1466.

(S3) Sphinx project, Welcome to the SPHInX repository. `https://sxrepo.mpie.de/`, Accessed: 2019-08-02.

(S4) Brivio, F.; Walker, A. B.; Walsh, A. Structural and electronic properties of hybrid perovskites for high-efficiency thin-film photovoltaics from first-principles. *APL Mater.* **2013**, *1*, 042111.

(S5) Skelton, J. M.; Burton, L. A.; Parker, S. C.; Walsh, A.; Kim, C.-E.; Soon, A.; Buckeridge, J.; Sokol, A. A.; Catlow, C. R. A.; Togo, A.; Tanaka, I. Anharmonicity in the high-temperature Cmcm phase of SnSe: soft modes and three-phonon interactions. *Phys. Rev. Lett.* **2016**, *117*, 075502.

(S6) Cohen, A. V.; Egger, D. A.; Rappe, A. M.; Kronik, L. Breakdown of the Static Picture of Defect Energetics in Halide Perovskites: The Case of the Br Vacancy in CsPbBr3. *J. Phys. Chem. Lett.* **2019**, *10*, 4490–4498.

(S7) Alkauskas, A.; Yan, Q.; Van de Walle, C. G. First-principles theory of nonradiative carrier capture via multiphonon emission. *Phys. Rev. B* **2014**, *90*, 075202.

(S8) Kim, S.; Hood, S. N.; van Gerwen, P.; Whalley, L. D.; Walsh, A. CarrierCapture.jl: Anharmonic Carrier Capture. *J. Open Source Softw.* **2020**, *5*, 2102.

(S9) Kim, S.; Hood, S. N.; Walsh, A. Anharmonic lattice relaxation during nonradiative carrier capture. *Phys. Rev. B* **2019**, *100*, 041202.

(S10) Barbary, K. Julia library for 1-d and 2-d splines. `https://github.com/kbarbary/Dierckx.jl`, Accessed: 2019-08-02.

(S11) Solve the N-D time-independent Schrödinger equation for a single particle. `https://github.com/RedPointyJackson/Brooglie`, Accessed: 2019-08-02.

(S12) Bystrom, K. Parallel C/Python package for numerical analysis of PAW DFT wavefunctions. https://github.com/kylebystrom/pawpyseed, Accessed: 2019-08-02.
